# Proposal for a new tool assessing validity performance in forensic neuropsychological testing: the Test of Malingering in Abstraction Skills (TOMAS)

**DOI:** 10.1007/s10072-025-08061-6

**Published:** 2025-03-03

**Authors:** Francesco Panico, Eleonora Fonzo, Annalisa Verde, Simona Lancia, Luigi Trojano

**Affiliations:** University of Campania “Luigi Vanvitelli”, Viale Ellittico 31, Caserta, 81100 Italy

**Keywords:** Malingering, Forensic, Neuropsychological assessment, Performance validity testing, Poor effort

## Abstract

**Objective:**

the assessment of malingering in forensic neuropsychological testing can be supported by the use of performance validity tests (PVTs). When designing PVTs, test material should be easy enough to be insensitive to real cognitive dysfunction, but at the same time difficult enough not to appear overtly as a measure of poor effort. In the present paper, we aimed at proposing a new instrument, the Test of Malingering in Abstraction Skills (TOMAS), for detecting poor effort possibly due to malingering in forensic neuropsychological assessment; in designing the instrument, we ensured that the test had a credible level of difficulty to keep satisfactory sensitivity.

**Method:**

the TOMAS was developed as a standalone tool utilising items selected from standardised and validated neuropsychological tests assessing verbal abstraction skills. In three studies we developed the final version of the test, assessed its association with demographic and cognitive variables, and estimated its sensitivity, specificity and criterion validity in comparison with the Rey 15-items test using a simulation paradigm involving healthy participants.

**Results:**

the final version of the TOMAS includes two sections (Part A and Part B) providing multiple indexes that have an adequate discriminating power, with satisfactory sensitivity and specificity values; the discriminating power of the TOMAS is higher than that of the Rey 15-items test.

**Conclusion:**

the multiple indexes provided by the TOMAS may support clinicians in assessing poor effort during neuropsychological examination. Future evidence is needed to fully establish the validity of the instrument in clinical and forensic samples.

**Supplementary Information:**

The online version contains supplementary material available at 10.1007/s10072-025-08061-6.

## Introduction

Forensic neuropsychology is concerned with the need to provide scientifically grounded answers to specific forensic questions related to cognitive functioning [1, 2]. It employs the knowledge and the methods of neuropsychology to support legal decision making [1]. Although a close relationship exists between clinical and forensic neuropsychology, some aspects define the peculiar boundaries of these specialities [2]. Indeed, in the clinical context the neuropsychologist is faced with a patient and their relatives representing the clients (and in this context rehabilitative purposes are usually prioritised), while in the forensic context the neuropsychologist is normally questioned by lawyers (without rehabilitative intentions). Another relevant difference between clinical and forensic neuropsychology is the specific need in the latter for estimating clients’ effort during the assessment process, which may inform about possible malingering. Indeed, because of the external incentives associated with the forensic question -for instance in terms of compensation following a work-related injury or mitigation of sentence to be ascribed to intellectual disability- the neuropsychologist must be concerned with the possibility that the examinees malinger. Malingering in neuropsychological evaluation can be defined as the intentional production of false or exaggerated cognitive deficits for the purpose of achieving identifiable external rewards [3]. During neuropsychological testing, a person who is malingering deliberately and intentionally underperforms to the aim of receiving benefits or avoiding prosecution for legal responsibility [3].

Given the consequences at the individual and collective levels of false-positive and false-negative conclusions, several methods have been proposed to support malingering assessment during forensic neuropsychological evaluation [2, 4, 5, 6]. Performance Validity Testing (PVT) refers to neuropsychologists’ methodology aimed at determining whether neuropsychological test performance is validly attributable to true neurocognitive (dys)function, or it is overtly impacted by the patient’s intent during the exam. PVTs can be either standalone tests designed for this sole purpose, or specific measures embedded within traditional neuropsychological tests [7]. Recent surveys in the United States, Canada and Europe have shown that neuropsychologists, although with different rates across countries, use a variety of measures for the purpose of assessing performance validity as part of everyday forensic and clinical practice [2, 8, 9, 10]. Moreover, the scientific base for PVT has expanded greatly over the last 20 to 30 years [11]. While the literature on PVT has warned about the risks of strongly relying on such instruments in determining malingering, and about statistical and methodological limitations in their use and development [12, 13], the combination of PVTs with other behavioural and qualitative indexes to assess adequate effort may complement recognition of malingering in the legal context [7, 11, 14].

When developing PVTs researchers are concerned with the need to achieve adequate *sensitivity*, namely the correct identification of malingerers, and adequate *specificity*, namely the correct identification of non-malingerers [7, 11, 12, 14]. To devise instruments possibly insensitive to the cognitive dysfunctions, one could opt to oversimplify test material at cost of reducing sensitivity [11, 15]. Here in contrast, with the aim to preserve sensitivity and avoid being easily recognisable as an effort test, we propose a new instrument, the Test of Malingering in Abstraction Skills (TOMAS), with a convincing level of difficulty. The TOMAS is a standalone instrument using items selected from validated neuropsychological tests examining verbal abstraction skills. The test is composed of two sections (Part A and Part B), with the Part B specifically developed to be easier than the Part A. However, the two sections are designed and introduced to the examinees in a way biasing their expectations toward the opposite direction. Thus, while in case of genuine performance an improvement is normally expected from Part A and B, in case of poor effort such improvement should be abolished. In the present paper we describe the procedure followed for the development of the test (Study 1), and provide data about sensitivity, specificity and criterion validity of the TOMAS (Studies 2 and 3). The multiple indexes provided by the TOMAS (chance level cut-offs, normative cut-offs, and a composite malingering index) are also described to support its possible application in research, clinical and forensic contexts.

## Study 1. Development of the TOMAS

The items used to build a preliminary version of the TOMAS were borrowed from the cognitive estimation tests by Nichelli [16] (20 items) and Della Sala [17] (21 items). Such instruments consist in simple orally presented questions on daily life issues -concerning for instance the weight of a pair of shoes or the time to have an egg cooked- which require examinees to produce a reasonably accurate estimate; examinee’s responses are scored with reference to the ranges of responses provided by healthy individuals in normative studies [16, 17].

In the construction of the two parts of the TOMAS, instead, we used the normative ranges of the original tests as multiple-choice alternatives from which the examinees are required to pick the most accurate estimate for each question. The 41 items belonging to the two original tests were used to obtain two parallel versions of the test for devising Part A and Part B of the preliminary version of the TOMAS. In the Part A, examinees are required to choose the correct response between just two possible answers, for inducing the erroneous idea that identifying the correct response is easy. However, here the wrong alternative differs slightly from the correct one, thus the Part A requires accurate estimates. Specifically, in this section of the test the alternatives corresponded to slightly or moderately inaccurate ranges (not considering odd responses) in Nichelli and Della Sala tests [16, 17]. In the Part B three alternative answers are provided, only one of which is correct. We reasoned that having three alternatives to choose among would have led the examinee to consider this part of the test more complex, although this is not the case because the correct response is definitely distinct from the two distractors, which corresponded to the ranges of very inaccurate and odd responses in normative studies for Nichelli and Della Sala tests [16, 17].

Once the two sets of items and alternatives were completed, 20 and 21 items were randomly shuffled to form Part A and Part B respectively. The position of the correct answer within each item was randomised and no item was repeated in both parts of the test.

In administering the TOMAS, the examiner introduces the test as tapping the ability to perform estimates in everyday life. The examiner also informs that the test comprises two parts, with increasing difficulty level (see the supplementary files for the test materials and their English translation).

### Participants

The preliminary version of the TOMAS (Parts A and B) was administered to a convenience sample of 66 healthy individuals (32 female), aged 18–82 years (M = 39.1, SD = 19.5) with levels of education ranging from “primary school” to “second level master’s degree or doctorate” (M = 12.3, SD = 3.41). All participants provided their written informed consent. This study, as well as the two next studies, was approved by the Ethics Committee of the Department of Psychology, University of Campania “Luigi Vanvitelli”, and was conducted following principles of the Helsinki Declaration and subsequent modifications.

### Statistical analyses

For each item, the total number of correct answers and percent accuracy over the total number of participants were calculated to identify possible tricky items. In selecting the items to be included in the final version of the TOMAS, a relevant concern was to make the test reasonably difficult, but not excessively challenging, and at the same time maximising the differences in complexity between the two parts of the instrument. For this reason, and in view of building the final version of the TOMAS, we chose to exclude from Part A the items with a percentage of correct answers lower than 40% and from Part B the items with an accuracy lower than 60% in the study sample.

Normality of data was explored by the Kolmogorov-Smirnov test to choose the appropriate statistics for comparing accuracy on Part A and B of this preliminary version of the TOMAS. Analyses were conducted using IBM SPSS Statistics v.20.

In the lack of specific literature to set the parameters for an a priori sample size estimation, we computed the achieved power of the analyses a posteriori using the G*Power software v. 3 [18], considering as adequate values of.80 or above [19].

## Results

Based on the item analysis showing a low percentage of correct responses, 2 items were removed from the TOMAS Part A. The Kolmogorov-Smirnov test showed a non-normal distribution of the data (*p* =.001). The non-parametric Wilcoxon paired-rank signed test showed that the percentage of correct answers in Part A (M = 61.1; SD = 12.1) was significantly lower than that in Part B (M = 71.5; SD = 12.6; Z= -5.28; *p* <.001), in line with our purposes.

Post-hoc power analysis revealed an adequate achieved power of.96 (input parameters: tails = 2, parent distribution = minARE, d =.5, alpha error probability =.05).

### Comment

Study 1 confirmed the desired properties of the TOMAS, being the items included in Part A with a two-choice format apparently easier, but in fact significantly harder, than those included in Part B (where three-choice response alternatives are given). This study allowed to define the items (*N* = 39) to be included in the final version of TOMAS. The final version was also slightly modified to make some response alternatives clearer and the whole test easier to read (e.g. by presenting units of measurements in extended forms, avoiding symbols and abbreviations). The optimised version of the TOMAS was used in the next stages of the research.

## Study 2. Testing the TOMAS structure and correlation with demographic variables

### Participants

The new version of the TOMAS was completed by a convenience sample of 83 healthy individuals (52 female; age: 20–85 years, M = 41.35, SD = 18.27; education: 5–17 years of formal education, M = 13.17, SD = 3.65) who did not take part to Study 1. All participants provided their written informed consent. The study was approved by the local Ethics Committee and was conducted following principles of the Helsinki Declaration, and subsequent modifications.

### Materials

The new version of the TOMAS obtained from Study 1 was administered (supplementary files), together with two neuropsychological tests aimed at obtaining a measure of general cognitive functioning and of abstraction skills: the Montreal Cognitive Assessment (MOCA) [20] and the Cognitive Estimation Test (CET) [21]. The MOCA is a brief neuropsychological tool for screening global cognitive functioning. The test provides a maximum score of 30; the higher the score, the higher the level of global cognitive efficiency. The CET explores the ability to perform abstract judgements on a range of everyday topics; scores range 0–27 with higher scores indicating lower cognitive estimation abilities. Raw scores on the two tests were corrected as a function of age and educational level.

### Statistical analyses

Percent accuracy on the two parts of the TOMAS was computed. Moreover, a malingering index was computed for each participant by subtracting the number of correct responses on Part A from the correct responses on Part B of the TOMAS (Part B-Part A). Normality of data was explored by the Kolmogorov-Smirnov test to choose the statistic to compare accuracy on Parts A and B of the TOMAS, and to search for associations of performance on Parts A and B, and of the malingering index, with the demographic variables and the measures of cognitive functioning.

Analyses were conducted using SPSS Statistics v.20.

A posteriori computation of the achieved power for each analysis was performed by means of G*Power software v. 3 [18], considering as adequate values of.80 or above [19].

## Results

Table 1 reports the descriptive statistics of the sample. The Kolmogorov-Smirnov test showed non-normality of the distribution of responses on Part A (*p* =.01) and Part B (*p* <.001) of the TOMAS, as well as of the computed malingering index (*p* <.001). Thus, non-parametric analyses were conducted. The Wilcoxon signed-rank test confirmed that accuracy at Part B of the TOMAS was significantly higher as compared to the one at Part A of the test (Z=-6.94, *p* <.001).

**Table 1:** Descriptive statistics of the scores obtained by the sample (*N* = 83)

	Mean	St. Dev.	Min	Max	Median
% TOMAS A	60.31	11.77	33.33	83.33	61.11
% TOMAS B	75.96	12.15	33.33	95.24	76.19
Mal Index	5.10	2.74	-4	12	5
MOCA corrected*	25.20	3.29	15.85	30.00	25.52
CET corrected*	10.89	3.91	3.45	22.45	10.45

Notes. TOMAS: Test of Malingering in Abstraction Skills; MOCA: Montreal Cognitive Assessment; CET: Cognitive Estimation Test; *for MOCA and CET raw data have been corrected as a function of age and education according to normative data

Then we computed Spearman correlations to assess possible associations of accuracy on TOMAS Part A and B, and of the malingering index, with years of age and of formal education, sex (coded as: “male” =1, “female” =0), and age- and education-corrected scores on MOCA and CET (Table 2). Accuracy at TOMAS Part A significantly and positively correlated with education. Accuracy at TOMAS Part B significantly and positively correlated with corrected MOCA score and significantly and negatively correlated with corrected CET score. The malingering index showed no correlation with any of the demographic variables or measures of cognitive functioning. Accuracy at the TOMAS Part A and B, as well as the malingering index, did not differ as a function of age and gender of participants.

**Table 2:** Spearman correlations (r) and levels of significance for the TOMAS scales, demographics, and neuropsychological measures

Spearman correlations	TOMAS A	TOMAS B	Mal Index	Sex	Age	Education	MOCA corrected	CET corrected
TOMAS A	1.00	**0.35** ^**^	**− 0.52** ^**^	0.11	0.03	**0.24** ^*^	0.15	-0.16
TOMAS B		1.00	**0.56** ^**^	0.18	-0.08	0.05	**0.31** ^**^	**− 0.24** ^*^
Mal Index			1.00	0.07	-0.08	-0.16	0.14	-0.12

Notes. TOMAS: Test of Malingering in Abstraction Skills; MOCA: Montreal Cognitive Assessment; CET: Cognitive Estimation Test

Post-hoc power analysis on the Wilcoxon signed-rank test revealed an adequate achieved power of.98 (input parameters: tails = 2, parent distribution = minARE, d =.5, alpha error probability =.05). Post-hoc power analysis for correlational analyses revealed a satisfactory achieved power of.80 (input parameters: tails = 2, *p*H1 =.3, *p*H0 = 0, alpha error probability =.05).

### Comment

The second study confirmed the desired main property of the TOMAS, i.e. that Part A was significantly harder than Part B, despite its apparently simpler format. Moreover, we observed that, although accuracy scores on Parts A and B were significantly correlated with each other and were variably correlated with education (Part A) or neuropsychological scores (Part B), the derived malingering index showed no association with demographics or measures of cognitive functioning. As a further step we ascertained whether the TOMAS was sensible to poor participants’ effort by conducting a third study using a simulation paradigm [11].

## Study 3. Sensitivity and specificity of the TOMAS

This study aims to assess sensitivity and specificity of the TOMAS in discriminating malingering, to provide initial information on criterion validity, and to obtain chance level and normative cut-offs. To this end, a simulation paradigm was implemented with two groups of healthy volunteers: a group of participants was instructed to simulate a cognitive deficit (instructed malingerers), and a group of participants was invited to provide their best performance (instructed non-malingerers) [22, 23]. Moreover, we compared the TOMAS with a well-known instrument commonly used to assess malingering, the Rey 15-item Test [24]. We expected that the participants assigned to the instructed non-malingerer group showed an improvement from Part A and Part B of the test, with a positive value of malingering index, while such a trend should be absent in the instructed malingering group. We also expected that the average percent accuracy in the instructed malingerer group was lower than in the instructed non-malingerer group. Finally, we expected that the greater overall difficulty of the items composing the TOMAS would restrict doubtfulness toward the test, increasing sensitivity of the TOMAS in detecting malingering as compared to the Rey’s 15-items test [24].

### Participants and experimental procedures

A sample of 87 participants (58 female) aged 20–69 years (M = 35.6; SD = 13.1) with different levels of education (5–18 years, M = 14.03, SD = 2.99), not recruited in the previous studies, voluntarily participated in the Study 3 provided their written consent. The sample size was based on the Receiving Operating Characteristic (ROC) analysis using the MedCalc^®^ Statistical Software version 23.1.3 (MedCalc Software Ltd, Ostend, Belgium; https://www.medcalc.org) with an estimated area under the curve of.75, an alpha value of.05, a beta value of.15, and a positive/negative ratio of 1, resulting in a minimum sample size of 44 participants. Participants were either university students or young workers of the public or private sectors recruited through examiners’ acquaintances or social media. The study was approved by the local Ethics Committee and was conducted following principles of the Helsinki Declaration, and subsequent modifications.

Participants were told that they would have completed a neuropsychological assessment focusing on their cognitive functioning. As a preliminary measure of global cognitive functioning, the MOCA [20] was administered to all participants before assigning them to the experimental groups, so to ascertain possible differences in general cognitive functioning. Subsequently, the participants were invited to draw from a box one of two scenarios (each depicting one experimental condition and identified by a numerical code) and to behave accordingly. After a few minutes the experimenter returned to the experimental room to administer the TOMAS and a short battery of tests (see below).

### Simulation paradigm

After the extraction of the scenario, participants were given a short time to get into the role (from 2 to 5 min). Then they were asked to put the scenario back in the box and to call the examiner back. The participants were warned not to tell the examiner the outcome of their draw; only at the end of the testing sessions the examiner asked the code associated with the scenario (not the content of the scenario). The examiner involved in the data collection was blind to the codes associated with each scenario (and hence to their content) for the entire time the data collection took place.

Full instructions describing the two scenarios are reported as supplementary files.

### Neuropsychological battery

The neuropsychological battery included the MOCA [20], the Frontal Assessment Battery (FAB) [25], the TOMAS, the CET [21] and the Rey 15-item Test [24].

The FAB [25] is a screening neuropsychological battery for evaluating executive functions; the maximum total score is 18, with higher scores indicating higher level of executive functioning.

The Rey 15-item Test [24] consists of a stimulus card containing 15 items to be reproduced by examinees upon card removal. Although the task may seem difficult because it contains 15 items, it is actually very simple because chunking of stimuli makes them very easy to remember also by individuals with memory impairments [26].

The tests were administered to all participants in the described order (MOCA, FAB, TOMAS, Rey 15-items), and only the MOCA was administered before assignment to the experimental group.

### Statistical analyses

Descriptive statistics for demographic variables, age- and education-corrected scores on the neuropsychological tests, total number of reproduced items at Rey 15-item test, total number of correct responses and accuracy on TOMAS Parts A and B, and the malingering index (Part B-Part A) were computed in the two groups.

To exclude differences in demographics and general cognitive functions, a one-way ANOVA was run on age, education and corrected scores at the MOCA test with the Group (instructed malingerers vs. instructed non-malingerers) as a between-subject factor.

Then we compared the groups on the accuracy rates at TOMAS Part A and B, on the malingering index, and on the Rey 15-item test after exploring normality by the Kolmogorov-Smirnov test [27].

ROC analysis was used to assess the ability of the TOMAS Parts A and B, of the malingering index, and of the Rey 15-item test, to distinguish between instructed malingerers and non-malingerers. Measures of sensitivity and specificity were calculated for the TOMAS Part A and B, for the malingering index, and for the Rey 15-item test. In case of a significant discriminative effect (*p* <.05), we calculated the cut-off scores using the Youden index (Sensitivity + Specificity – 1) [28]. Areas Under the Curve (AUCs) values of the TOMAS and of 15-items Rey were compared using DeLong’s test [29].

Finally, chance-level cut-offs were calculated using the binomial distribution considering the total number of items (18 for TOMAS Part A, 21 for TOMAS Part B) and the probability of correct responses by chance at each item (.05 for TOMAS Part A,.03 for TOMAS Part B).

Statistical analyses were run in SPSS v.20 and Jamovi v. 2.3.28.

## Results

The two experimental groups did not differ for age [F(1,85) = 1.09, *p* =.29, η²=.01], education [F(1,85) =.95, *p* =.33, η²=.01] or general cognitive functioning [F(1,85) = 2.55, *p* =.11, η²=.03] (Table 3).

**Table 3:** Descriptive statistics of the study variables for the two experimental groups

	Instructed Malingerers (*N* = 41)			Instructed non-Malingerers (*N* = 46)		
	Mean	St. Dev.	Range	Mean	St. Dev.	Range
Age	34.02	12.26	21–69	36.96	13.76	20–68
Education	14.37	2.41	8–18	13.74	3.44	5–18
MOCA corrected	24.03	2.37	16.59–27.59	25.06	3.49	15.85-30
% TOMAS A	55.83	13.72	33.33–83.33	59.78	12.68	33.33–83.33
% TOMAS B	53.82	19.64	4.76–85.71	77.31	13.17	33.33–95.24
N. correct TOMAS A	10.05	2.46	6–15	10.76	2.28	6–15
N. correct TOMAS B	11.32	4.13	1–18	16.24	2.77	7–20
Mal Index	1.27	3.27	-7-8	5.48	2.38	-4-10
FAB corrected	7.65	4.73	1.4–15.7	15.45	2.54	7.7–18
CET corrected	13.56	4.58	6.62–23.45	10.39	4.89	3.45–20.45
15-Item Rey	12.46	3.26	2–15	14.00	2.16	6–15

Notes. TOMAS: Test of Malingering in Abstraction Skills; MOCA: Montreal Cognitive Assessment; FAB: Frontal Assessment Battery; CET: Cognitive Estimation Test

The Kolmogorov-Smirnov test showed that measures were not normally distributed (*p* <.05) thus conservative non-parametric methods were used. Mann-Whitney test showed no difference between the groups in the accuracy on TOMAS Part A (U = 775, *p* =.15), but a significant difference was found in the accuracy on TOMAS Part B (U = 283.5, *p* <.001) and in the malingering index (U = 254.5, *p* <.001). Moreover, the Wilcoxon test demonstrated that while non-malingerers showed the previously described increase in accuracy from Part A to Part B of the TOMAS (Z=-5.43, *p* <.001), this trend was absent in the malingerer group (Z=-0.38, *p* =.71). Moreover, statistical analyses showed a higher number of reproduced items on Rey 15-item test in non-malingerers as compared to malingerers (U = 648, *p* =.004).

**Fig. 1:**
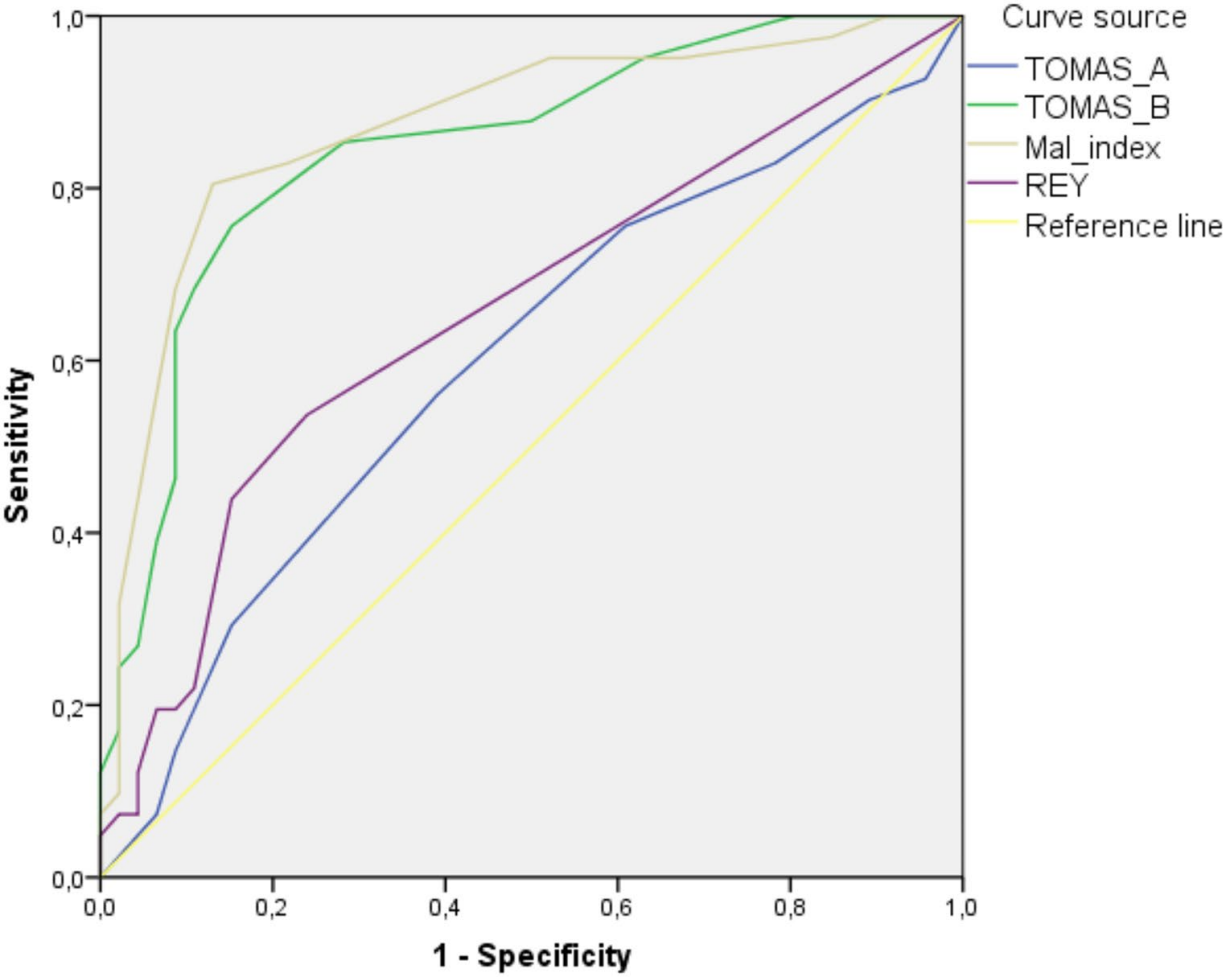
Receiving Operating Characteristic curve for the TOMAS Part A and B, the malingering index and the Rey 15-item test in instructed non-malingerers and instructed malingerers groups

The ROC analysis showed good discriminating performance in detecting instructed malingerers versus instructed non-malingerers for both the total scores at the TOMAS Part B and the malingering index (TOMAS Part B: AUC =.85, *p* <.001, SE =.04, CI 95%=.77–.93; TOMAS Mal Index: AUC =.87, *p* <.001, SE =.04, CI 95%=.79–.95; Fig. 1). TOMAS Part A did not show valuable discriminant power (AUC =.60, *p* =.12). The Rey 15-item test showed a significant discriminant performance although with lower AUC (AUC =.66, *p* =.012, SE =.06, CI 95%=.54-.77) with respect to TOMAS Part B score and the malingering index.

Table 4 describes the specificity, sensitivity for multiple cut-offs of the TOMAS Part B and of the malingering index for Youden’s Indexes above.50. Youden Index values suggested a cut-off of 14/21 correct responses for TOMAS Part B (Youden’s Index =.60), and a cut-off of 3 for the malingering index (Youden’s Index =.67). As far as the Rey 15-item test is concerned the maximum value of the Youden’s Index calculated on our sample was.297, corresponding to a cut-off of 13/15 with modest specificity (.76) and low sensitivity (.54).

**Table 4:** Best discriminant cut-offs of the TOMAS between instructed malingerers and non-malingerers groups

TOMAS Part B			
Score	Sensitivity	Specificity	Youden’s Index
12	0.63	0.91	0.55
13	0.68	0.89	0.57
14	0.76	0.85	0.60
15	0.85	0.72	0.57
Malingering Index			
Score	Sensitivity	Specificity	Youden’s Index
2	0.68	0.91	0.60
3	0.80	0.87	0.67
4	0.83	0.78	0.61

DeLong tests showed that AUCs of the TOMAS Part B and of the Malingering Index were significantly higher than the one of Part A and of the Rey 15-item test (Table 5).

**Table 5:** DeLong test of differences between AUCs

Estimated AUC’s					
	AUC	SD(Hanley)	*P*(H0: AUC=0.5)	SD(DeLong)	*P*(H0:AUC=0.5)
TOMAS Part A	0.598	0.061	0.055	0.061	0.055
TOMAS Part B	0.849	0.043	0.0	0.042	0.0
Mal Index	0.872	0.040	0.0	0.040	0.0
Rey 15-items	0.656	0.059	0.004	0.052	0.001
Pairwise comparisons					
Variable	AUC Difference	CI(lower)	CI(upper)	p value	Correlation
TOMAS Part A vs. Part B	-0.251	-0.355	-0.147	0.001	0.529
TOMAS Part A vs. Mal Index	-0.275	-0.423	-0.126	0.001	-0.083
TOMAS Part A vs. Rey 15-items	-0.059	-0.215	0.098	0.464	0.015
TOMAS Part B vs. Mal Index	-0.024	-0.093	0.046	0.501	0.626
TOMAS Part B vs. Rey 15-items	0.192	0.065	0.320	0.003	0.060
Mal Index vs. Rey 15-items	0.216	0.098	0.334	0.001	0.163
Overall test					
p-value< 0.001					

Chance-level cut-offs calculated using binomial distribution yielded a cut-off of 5 for the TOMAS Part A (*p* =.033), and a cut-off of 3 for the TOMAS Part B (*p* =.035).

### Comment

Study 3 used a simulation paradigm to assess the capability of the TOMAS to discriminate instructed malingerers from non-malingerers. The results showed that Part A of the TOMAS was largely insensitive to malingering, whereas the Part B of the TOMAS and the malingering index have an adequate discriminating power. Relevantly, the TOMAS showed higher sensitivity and significantly larger AUC values compared with the Rey-15 items test.

## Discussion

Forensic neuropsychology has shown a remarkable growth from the 1990s to current times. Almost 50% of the respondents to a recent survey in the United States of America and Canada engaged in forensic neuropsychology practice in 2020 [2]. A similar percentage was obtained in Europe, where the interest in forensic neuropsychology is increasing in more recent years [9]. Crucially, even though most of the respondents to that survey reported general knowledge about symptom validity in forensic neuropsychological assessment, a sizeable proportion of them relied on outdated notions, e.g. that clinicians can determine symptom credibility based on intuitive judgment, with large differences on use of PVTs in their professional practice. As discussed in recent authoritative papers on the topic, new criteria, instruments and methodology to assess malingering are needed to develop the discipline further [7, 11, 12, 13, 14].

In the present paper, we propose a new tool for supporting the assessment of malingering in forensic neuropsychological evaluation. The TOMAS has been designed as a standalone test to assess poor effort possibly due to malingering asking examinees to answer questions about daily-life issues. The development of the TOMAS was intended to have a moderate level of difficulty to avoid being considered a test assessing compliance with the testing process. Here we also provided data confirming robustness of the test structure, capability to assess poor effort, and cut-offs to orient the decision of forensic neuropsychologists in its use.

The interpretation of performance on the TOMAS is based on multiple indexes. First, cut-offs of the TOMAS Part B score and the malingering index obtained from the normative sample can be used to detect invalid performance. A score falling below the normative cut-offs (14/21 on TOMAS Part B and 3 on the malingering index) might be considered as an indicator of poor effort. Chance level cut-offs suggest a performance lower than 5/18 on TOMAS Part A and lower than 3/21 on TOMAS Part B to be significantly unlikely by chance, possibly indicating that examinees have identified the right response but intentionally provided the wrong one. Examinees’ performance falling between normative and chance level cut-off should raise particular attention during forensic neuropsychological assessment. Nevertheless, it should be noted that the assessment of malingering is a multidimensional process, which does not rely exclusively in administering and interpreting PVTs, as it has been largely discussed elsewhere [14]. Indeed, assessment of malingering requires the inclusion and exclusion of external incentives, unreliable manifestations of symptoms, and incongruence in the overall clinical pictures. Moreover, it is generally recommended to include more than one PVTs during assessment, carefully exploring performance coherence [7, 11, 14].

The results obtained here should be considered preliminary and propaedeutic for validation studies. First, although we provided multiple indexes to detect possible malingering attitudes, it is essential to test the instrument in diverse neurological populations to obtain normative clinical data. Indeed, to obtain solid information on PVTs is important to gather normative values also in clinical populations to demonstrate that the measurement obtained by using the instrument is relatively insensitive to cognitive defects [7, 14]. The verbal nature of the test makes it not suitable to be used in patients with language impairments or with clinical dementia. However, the TOMAS could provide useful insights in the assessment of more subtle malingering attempts in pretended outcomes of mild traumatic brain injury, or mild cognitive impairment. Therefore, in development of the TOMAS these neurological populations should be at the forefront of further validation studies. It would be also necessary to obtain normative data from participants recruited in forensic, or medico-legal contexts using a “known group” experimental protocol [11].

Second, here we used the Rey 15-items test for assessing convergent validity, as this test is frequently used in forensic settings for its rapidity of administration and appropriate specificity values. However, it is important to underline that convergent validity of the TOMAS should be further explored using other solid instruments to detect poor effort, such as the Test Of Memory Malingering [30].

In conclusion, the TOMAS, with its sensitivity and specificity values and cut-offs for interpretation of performance, could reveal useful to support the clinicians in assessing poor effort possibly due to malingering in neuropsychological examination.

## Electronic supplementary material

Below is the link to the electronic supplementary material.

Supplementary Material 1

Supplementary Material 2

Supplementary Material 3

Supplementary Material 4

Supplementary Material 5

## Data Availability

Data will be shared upon request to the corresponding author.

## References

[1] Hom J (2003) Forensic neuropsychology: are we there yet? Arch Clin Neuropsychol 18:827–845. 10.1016/S0887-6177(03)00076-3 14609579 10.1016/s0887-6177(03)00076-3

[2] Sweet JJ, Boone KB, Denney RL et al (2023) Forensic neuropsychology: history and current status. Clin Neuropsychol 37:459–474. 10.1080/13854046.2022.2078740 35658794 10.1080/13854046.2022.2078740

[3] Iverson GL (2006) Ethical issues associated with the assessment of exaggeration, poor effort, and malingering. Appl Neuropsychol 13:77–90. 10.1207/s15324826an1302_3 17009881 10.1207/s15324826an1302_3

[4] Faust D (2023) Invited Commentary: advancing but not yet Advanced: Assessment of Effort/Malingering in forensic and clinical settings. Neuropsychol Rev 33:628–642. 10.1007/s11065-023-09605-3 37594693 10.1007/s11065-023-09605-3

[5] Sweet JJ, Heilbronner RL, Morgan JE et al (2021) American Academy of Clinical Neuropsychology (AACN) 2021 consensus statement on validity assessment: Update of the 2009 AACN consensus conference statement on neuropsychological assessment of effort, response bias, and malingering. Clin Neuropsychol 35:1053–1106. 10.1080/13854046.2021.1896036 10.1080/13854046.2021.1896036 33823750

[6] Marcopulos BA, Kaufmann P, Patel AC (2024) Forensic neuropsychological assessment. Behav Sci Law 1–13. 10.1002/bsl.2656 10.1002/bsl.2656 38583136

[7] Greher MR, Wodushek TR (2017) Performance validity testing in neuropsychology: scientific basis and clinical application - A brief review. J Psychiatr Pract 23:134–140. 10.1097/PRA.0000000000000218 28291039 10.1097/PRA.0000000000000218

[8] Brooks BL, Ploetz DM, Kirkwood MW (2016) A survey of neuropsychologists’ use of validity tests with children and adolescents. Child Neuropsychol 22:1001–1020. 10.1080/09297049.2015.1075491 26295363 10.1080/09297049.2015.1075491

[9] Dandachi-FitzGerald B, Ponds RWHM, Merten T (2013) Symptom validity and neuropsychological assessment: a survey of practices and beliefs of neuropsychologists in six European countries. Arch Clin Neuropsychol 28:771–783. 10.1093/arclin/act073 24047545 10.1093/arclin/act073

[10] LaDuke C, Barr W, Brodale DL, Rabin LA (2018) Toward generally accepted forensic assessment practices among clinical neuropsychologists: a survey of professional practice and common test use*. Clin Neuropsychol 32:145–164. 10.1080/13854046.2017.1346711 28675974 10.1080/13854046.2017.1346711

[11] Wodushek TR, Greher MR (2017) Performance validity testing in neuropsychology: methods for measurement development and maximizing diagnostic accuracy. J Psychiatr Pract 23:214–220. 10.1097/PRA.0000000000000233 28492460 10.1097/PRA.0000000000000233

[12] Leonhard C (2023) Review of statistical and methodological issues in the forensic prediction of malingering from Validity tests: part I: statistical issues. Neuropsychol Rev 33:581–603. 10.1007/S11065-023-09601-7 37612531 10.1007/s11065-023-09601-7

[13] Leonhard C (2023) Review of statistical and methodological issues in the forensic prediction of malingering from Validity tests: part II-Methodological issues. Neuropsychol Rev 33:604–623. 10.1007/S11065-023-09602-6 37594690 10.1007/s11065-023-09602-6

[14] Sherman EMS, Slick DJ, Iverson GL (2020) Multidimensional Malingering Criteria for Neuropsychological Assessment: a 20-Year update of the Malingered Neuropsychological Dysfunction Criteria. Arch Clin Neuropsychol 35:735–764. 10.1093/ARCLIN/ACAA019 32377667 10.1093/arclin/acaa019 PMC7452950

[15] Young G, Erdodi L, Giromini L, Rogers R (2024) Malingering-Related Assessments in Psychological Injury: Performance Validity Tests (PVTs), Symptom Validity Tests (SVTs), and Invalid Response Set. Psychol Inj Law 2024 1–16. 10.1007/S12207-024-09523-6

[16] Nichelli P, Leone M, Caronna A et al (2002) Taratura Di Un test di stime cognitive di impiego diagnostico in clinica: stime dei tempi e dei pesi (STEP). Nuova Riv Di Neurol 12:37–42

[17] Della Sala S, MacPherson SE, Phillips LH et al (2003) How many camels are there in Italy? Cognitive estimates standardised on the Italian population. Neurol Sci 24:10–15. 10.1007/S100720300015 12754651 10.1007/s100720300015

[18] Faul F, Erdfelder E, Lang AG, Buchner A (2007) G*Power 3: a flexible statistical power analysis program for the social, behavioral, and biomedical sciences. Behav Res Methods 39:175–191. 10.3758/bf03193146 10.3758/bf03193146 17695343

[19] Cohen J (1988) Statistical power analysis for the behavioural sciences, 2nd ed. Erlbaum

[20] Santangelo G, Siciliano M, Pedone R et al (2015) Normative data for the Montreal Cognitive Assessment in an Italian population sample. Neurol Sci 36:585–591. 10.1007/s10072-014-1995-y 25380622 10.1007/s10072-014-1995-y

[21] Scarpina F, D’Aniello GE, Mauro A et al (2015) How many segments are there in an orange: normative data for the new Cognitive Estimation Task in an Italian population. Neurol Sci 36:1889–1895. 10.1007/S10072-015-2276-0 26067453 10.1007/s10072-015-2276-0

[22] Hoover S, Zottoli TM, Grose-Fifer J (2014) ERP correlates of malingered executive dysfunction. Int J Psychophysiol 91:139–146. 10.1016/J.IJPSYCHO.2013.12.009 24394183 10.1016/j.ijpsycho.2013.12.009

[23] Lindstrom W, Coleman C, Thomassin K et al (2011) Simulated dyslexia in postsecondary students: description and detection using embedded validity indicators. Clin Neuropsychol 25:302–322. 10.1080/13854046.2010.537280 21184348 10.1080/13854046.2010.537280

[24] Rey A (1964) The clinical examination in psychology. University Press of France, Paris

[25] Appollonio I, Leone M, Isella V et al (2005) The frontal assessment battery (FAB): normative values in an Italian population sample. Neurol Sci 26:108–116. 10.1007/s10072-005-0443-4 15995827 10.1007/s10072-005-0443-4

[26] Reznek L (2005) The Rey 15-item memory test for malingering: a meta-analysis. Brain Inj 19:539–543. 10.1080/02699050400005242 16134741 10.1080/02699050400005242

[27] Mishra P, Pandey CM, Singh U et al (2019) Descriptive statistics and normality tests for statistical data. Ann Card Anaesth 22:67–72. 10.4103/ACA.ACA_157_18 30648682 10.4103/aca.ACA_157_18 PMC6350423

[28] Berrar D (2018) Performance measures for binary classification. Encycl Bioinforma Comput Biol ABC Bioinforma 1–3:546–560. 10.1016/B978-0-12-809633-8.20351-8

[29] DeLong E, DeLong D, Clarke-Pearson D (1988) Comparing the areas under two or more correlated receiver operating characteristic curves: a nonparametric approach. Biometrics 44:837–845 3203132

[30] Tombaugh TN (1997) The test of memory malingering (TOMM): normative data from cognitively intact and cognitively impaired individuals. Psychol Assess 9:260

