# Supplementary material for: Proposal for a new tool assessing validity performance in forensic neuropsychological testing: the Test of Malingering in Abstraction Skills (TOMAS)
Source: Neurol Sci. 2025 Mar 3;46(6):2591–600. doi: 10.1007/s10072-025-08061-6 (PMC12084168; doi:10.1007/s10072-025-08061-6)
Supplement: Supplementary file 1 — Supplementary Material 1 [file 10072_2025_8061_MOESM1_ESM.docx]

**Supplementary file**

**Proposal for a new tool assessing validity performance in forensic neuropsychological testing: the Test of Malingering in Abstraction Skills (TOMAS)**

Francesco Panico, Eleonora Fonzo, Annalisa Verde, Simona Lancia, Luigi Trojano

*University of Campania “Luigi Vanvitelli”, Viale Ellittico 31, 81100 Caserta, Italy*

Content of the scenarios used in the simulation study (Study 3):

Scenario 1 (non-malingering): “*Imagine you are a victim of a car accident in which you hit your head. Luckily, you did not suffer any type of damage or injury from the accident, and you are not interested in any external financial incentives you might obtain. Now you are going to undergo an assessment of your cognitive functioning in which you will attempt at giving your best performance*".

Scenario 2 (malingering): “*Imagine you are a victim of a car accident in which you hit your head. Hitting the head violently can lead to memory, attention and reasoning troubles. You are going to undergo an assessment of your cognitive functioning, and you are determined to feign a cognitive deficit with the aim of obtaining a financial incentive. It is crucial that you consider that a performance characterized by major exaggerations can be easy to identify. Therefore, try to be convincing during the assessment*”.
