# Supplementary material for: Proposal for a new tool assessing validity performance in forensic neuropsychological testing: the Test of Malingering in Abstraction Skills (TOMAS)
Source: Neurol Sci. 2025 Mar 3;46(6):2591–600. doi: 10.1007/s10072-025-08061-6 (PMC12084168; doi:10.1007/s10072-025-08061-6)
Supplement: Supplementary file 2 — Supplementary Material 2 [file 10072_2025_8061_MOESM2_ESM.pdf]

Istruzioni TOMAS

Presentazione generale. Questo test valuta la tua capacità di eseguire stime accurate su alcuni aspetti di vita quotidiana. Si compone di due parti (A e B) caratterizzate da un livello crescente di difficoltà.

[l'esaminatore procede a presentare la prima parte dello strumento posizionando il plico contenente il materiale di somministrazione di fronte all'esaminando; vengono poi fornite le istruzioni specifiche per la Parte A. L'esaminatore prende nota delle risposte dell'esaminando utilizzando il foglio di notazione]

Parte A. Qui di seguito sono riportate alcune domande che richiedono di formulare una stima accurata. Dovrai scegliere la risposta corretta tra due alternative, di cui una sola è quella corretta, cercando di essere accurato nella tua stima.

[a conclusione della prima parte del test, senza fornire alcun riscontro sulla prestazione, l'esaminatore procede a fornire le istruzioni specifiche per la Parte B]

Parte B. Anche in questa parte del test verranno presentate delle domande su attività quotidiane. In questo caso il livello di difficoltà sarà maggiore in quanto dovrai scegliere la risposta giusta tra tre alternative, di cui una sola è quella giusta, facendo una stima quanto più possibile accurata.

[l'esaminatore procede a presentare uno ad uno i restanti quesiti della Parte B fino a conclusione del test; non sono previsti aiuti o chiarimenti. L'esaminatore prende nota delle risposte dell'esaminando utilizzando il foglio di notazione]

Parte A

Item	Alternative	Risposta Corretta	Risposta fornita	Punteggio 0\1
1. Qual è il peso di una saponetta?	A. Da 46 a 65 grammi<br>B. Da 66 a 150 grammi	B		
2. Quanto tempo ci si mette a graffettare 10 copie di 3 pagine di giornale?	A. Da 1 a 4 minuti<br>B. Da 5 a 6 minuti	A		
3. Quanto tempo ci mette una lavatrice a completare il programma di lavaggio per le lenzuola?	A. Da 41 minuti a 2 ore<br>B. Da 30 a 40 minuti	A		
4. Quanto tempo ci si mette a chiudere, scrivere l'indirizzo, e a mettere il francobollo su 5 lettere?	A. Da 2 a 4 minuti<br>B. Da 5 a 10 minuti	B		
5. Quanti cammelli ci sono in Italia?	A. Da 4 a 27<br>B. Da 28 a 52	B		
6. Qual è il peso di un acquario di 90 per 45 per 60 cm (senz'acqua)?	A. Da 3 a 9 kilogrammi<br>B. Da 1 a 2 kilogrammi	A		
7. Quanto ci si mette a cucire un bottone su una camicia?	A. Da 1 a 2 minuti<br>B. Da 3 a 5 minuti	B		
8. Quanto pesano delle scarpe con il tacco?	A. Da 280 a 520 grammi<br>B. Da 112 a 279 grammi	A		
9. Quanto è lunga mediamente la colonna vertebrale di un uomo?	A. Da 49 a 91 centimetri<br>B. Da 92 a 133 centimetri	A		
10. Qual è il peso dello specchietto retrovisore di un'automobile (quello interno)?	A. Da 401 a 650 grammi<br>B. Da 151 a 400 grammi	B		
11.Qual è il peso di una sedia del tavolo da pranzo?	A. Da 6 a 8 kilogrammi<br>B. Da 2 a 5 kilogrammi	B		
12. Quanto tempo ci mette un uomo a farsi fare uno shampoo e un taglio completo di capelli?	A. Da 31 a 55 minuti<br>B. Da 20 a 30 minuti	B		
13. Quanta acqua ci vuole per riempire una vasca da bagno?	A. Da 192 a 356 litri<br>B. Da 357 a 523 litri	A		
14. Qual è la lunghezza massima della Sicilia in Km?	A. Da 210 a 390 kilometri<br>B. Da 30 a 209 kilometri	A		
15. Quanto pesa un cavallo?	A. Da 280 a 520 kilogrammi<br>B. Da 521 a 760 kilogrammi	A		
16. Qual è il peso del paraurti posteriore di un'utilitaria (ad es. una panda)?	A. Da 1 a 2 kilogrammi<br>B. Da 3 a 9 kilogrammi	B		
17. Qual è la capacità di posti a sedere su un treno di 10 carrozze?	A. Da 504 a 936<br>B. Da 103 a 503	A		
18. Approssimativamente quanti caffè fa in un'ora il barista di un autogrill di un'autostrada nell'ora di punta?	A. Da 105 a 195<br>B. Da 47 a 104	A		

Parte B

Item	Alternative	Risposta Corretta	Risposta fornita	Punteggio 0\1
1. Qual è il peso di un asciugacapelli (di uso comune)?	A. Da 101 a 200 grammi<br>B. Da 301 a 550 grammi<br>C. Da 1 a 2 kilogrammi	B		
2. Quanti grammi di pasta devi cuocere per 4 persone?	A. Da 760 a 800 grammi<br>B. Da 1 a 40 grammi<br>C. Da 280 a 520 grammi	C		
3. Quante persone ci sono in un bus durante l'ora di punta?	A. Da 171 a 200<br>B. Da 63 a 117<br>C. Da 1 a 9	B		
4. Quanto impiega un uomo giovane per percorrere a piedi un km?	A. Da 7 a 13 minuti<br>B. Da 1 a 60 secondi<br>C. Da 19 a 20 minuti	A		
5. Quant'è lunga una carrozza passeggeri di un treno?	A. Da 17 a 33 metri<br>B. Da 1 a 2 metri<br>C. Da 48 a 50 metri	A		
6. Quanto tempo ci si mette a farsi una doccia?	A. Da 2 a 5 minuti<br>B. Da 9 a 15 minuti<br>C. Da 30 a 31 minuti	B		
7. Quanto pesa una maglietta di cotone da uomo?	A. Da 154 a 286 grammi<br>B. Da 1 a 22 grammi<br>C. Da 418 a 500 grammi	A		
8. Quanto tempo ci mette il caffè ad uscire da una caffettiera a due tazze?	A. Da 8 minuti a 3 ore<br>B. Da 7 secondi a 1 minuto<br>C. Da 2 a 5 minuti	C		
9. Quanti fiammiferi ci sono in una scatola?	A. Da 190 a 200<br>B. Da 1 a 10<br>C. Da 70 a 130	C		
10. Quanto tempo ci metti ad aspettare che il semaforo diventi verde?	A. Da 19 a 30 secondi<br>B. Da 1 a 3 minuti<br>C. Da 5 a 15 minuti	B		
11.Quant'è la superficie di un lenzuolo a due piazze?	A. Da 2 a 5 metri^2<br>B. Da 0 a 1 metri^2<br>C. Da 6 a 7 metri^2	A		
12. Quante settimane ci sono in un anno?	A. Da 99 a 120<br>B. Da 1 a 5<br>C. Da 36 a 68	C		
13. Qual è il peso di un paio di pantaloni di media taglia (tipo blue-jeans)?	A. Da 301 a 700 grammi<br>B. Da 0 a 100 grammi<br>C. Da 1 a 2 kilogrammi	A		
14. Quante ciglia ci sono nella palpebra inferiore?	A. Da 114 a 160<br>B. Da 0 a 6<br>C. Da 42 a 78	C		

15. Dopo che l’acqua bolle qual è il tempo necessario per cucinare un uovo sodo?	A. Da 1 a 15 secondi<br>B. Da 5 a 10 minuti<br>C. Da 15 a 20 minuti	B		
19. Qual è il peso di un coniglio (domestico)?	A. Da 3 a 8 kilogrammi<br>B. Da 101 grammi a 2 kilogrammi<br>C. Da 15 a 20 kilogrammi	A		
17. Quanto è alto un semaforo (pedonale)?	A. Da 5 a 8 metri<br>B. Da 0 a 1 metri<br>C. Da 2 a 4 metri	C		
18. Quanto tempo ci metti ad essere servito al banco del salumiere quando ci sono due persone prima di te?	A. Da 20 minuti a 3 ore<br>B. Da 10 a 15 minuti<br>C. Da 13 secondi a 5 minuti	B		
16. Quanto è veloce una rondine in km\h?	A. Da 56 a 104 kilometri/orari<br>B. Da 1 a 8 kilometri/orari<br>C. Da 152 a 160 kilometri/orari	A		
20. Quanto tempo ci metti ad allacciarti entrambe le scarpe?	A. Da 0 a 2 secondi<br>B. Da 18 secondi a 2 minuti<br>C. Da 4 a 10 minuti	B		
21. Qual è il peso di un paio di scarpe eleganti?	A. Da 1 a 2 kilogrammi<br>B. Da 101 a 200 grammi<br>C. Da 401 a 800 grammi	C		
