# Supplementary material for: Proposal for a new tool assessing validity performance in forensic neuropsychological testing: the Test of Malingering in Abstraction Skills (TOMAS)
Source: Neurol Sci. 2025 Mar 3;46(6):2591–600. doi: 10.1007/s10072-025-08061-6 (PMC12084168; doi:10.1007/s10072-025-08061-6)
Supplement: Supplementary file 3 — Supplementary Material 3 [file 10072_2025_8061_MOESM3_ESM.pdf]

Test Of Malingering in Abstraction Skills

Indicazioni per la stampa: stampare e rilegare i quesiti contenuti nel materiale di somministrazione su fogli formato A4 con orientamento orizzontale.

TOMAS

PARTE A

Qual è il peso di una saponetta?

A. Da 46 a 65 grammi

B. Da 66 a 150 grammi

Quanto tempo ci si mette a graffettare 10 copie di 3 pagine di giornale?

A. Da 1 a 4 minuti

B. Da 5 a 6 minuti

Quanto tempo ci mette una lavatrice a completare il programma di lavaggio per le lenzuola?

A. Da 41 minuti a 2 ore

B. Da 30 a 40 minuti

Quanto tempo ci si mette a chiudere, scrivere l'indirizzo, e a mettere il francobollo su 5 lettere?

A. Da 2 a 4 minuti

B. Da 5 a 10 minuti

Quanti cammelli ci sono in Italia?

A. Da 4 a 27

B. Da 28 a 52

Qual è il peso di un acquario di 90 per 45 per 60 cm (senz’acqua)?

A. Da 3 a 9 kilogrammi
B. Da 1 a 2 kilogrammi

Quanto tempo ci si mette a cucire un bottone su una camicia?

A. Da 1 a 2 minuti

B. Da 3 a 5 minuti

Quanto pesano delle scarpe col tacco?

A. Da 280 a 520 grammi
B. Da 112 a 279 grammi

Quanto è lunga mediamente la colonna vertebrale di un uomo?

A. Da 49 a 91 centimetri

B. Da 92 a 133 centimetri

Qual è il peso dello specchietto retrovisore di un’automobile (quello interno)?

A. Da 401 a 650 grammi

B. Da 151 a 400 grammi

Qual è il peso di una sedia del tavolo da pranzo?

A. Da 6 a 8 kilogrammi

B. Da 2 a 5 kilogrammi

Quanto tempo ci mette un uomo a farsi fare uno shampoo e un taglio completo di capelli?

A. Da 31 a 55 minuti

B. Da 20 a 30 minuti

Quanta acqua ci vuole per riempire una vasca da bagno?

A. Da 192 a 356 litri

B. Da 357 a 523 litri

Qual è la lunghezza massima della Sicilia in kilometri?

A. Da 210 a 390 kilometri

B. Da 30 a 209 kilometri

Quanto pesa un cavallo?

A. Da 280 a 520 kilogrammi

B. Da 521 a 760 kilogrammi

Qual è il peso del paraurti posteriore di un’utilitaria (ad es. una panda)?

A. Da 1 a 2 kilogrammi
B. Da 3 a 9 kilogrammi

Qual è la capacità di posti a sedere su un treno di 10 carrozze?

A. Da 504 a 936

B. Da 103 a 503

Approssimativamente quanti caffè fa in un’ora il barista di un autogrill di un’autostrada nell’ora di punta?

A. Da 105 a 195

B. Da 47 a 104

TOMAS

PARTE B

Qual è il peso di un asciugacapelli (di uso comune)?

A. Da 101 a 200 grammi
B. Da 301 a 550 grammi
C. Da 1 a 2 kilogrammi

Quanti grammi di pasta devi cuocere per 4 persone?

A. Da 760 a 800 grammi

B. Da 1 a 40 grammi

C. Da 280 a 520 grammi

Quante persone ci sono in un bus durante l’ora di punta?

A. Da 171 a 200
B. Da 63 a 117
C. Da 1 a 9

Quanto impiega un uomo giovane per percorrere a piedi un kilometro?

A. Da 7 a 13 minuti

B. Da 1 a 60 secondi

C. Da 19 a 20 minuti

Quant'è lunga una carrozza passeggeri di un treno?

A. Da 17 a 33 metri

B. Da 1 a 2 metri

C. Da 48 a 50 metri

Quanto tempo ci si mette a farsi una doccia?

A. Da 2 a 5 minuti
B. Da 9 a 15 minuti
C. Da 30 a 31 minuti

Quanto pesa una maglietta di cotone da uomo?

A. Da 154 a 286 grammi

B. Da 1 a 22 grammi

C. Da 418 a 500 grammi

Quanto tempo ci mette il caffè ad uscire da una caffettiera a due tazze?

A. Da 8 minuti a 3 ore
B. Da 7 secondi a 1 minuto
C. Da 2 a 5 minuti

Quanti fiammiferi ci sono in una scatola?

A. Da 190 a 200
B. Da 1 a 10
C. Da 70 a 130

Quanto tempo ci metti ad aspettare che il semaforo diventi verde?

A. Da 19 a 30 secondi

B. Da 1 a 3 minuti

C. Da 5 a 15 minuti

Quant’è la superficie di un lenzuolo a due piazze?

A. Da 2 a 5 metri^2
B. Da 0 a 1 metri^2
C. Da 6 a 7 metri^2

Quante settimane ci sono in un anno?

A. Da 99 a 120

B. Da 1 a 5

C. Da 36 a 68

Qual è il peso di un paio di pantaloni di media taglia (tipo blue-jeans)?

A. Da 301 a 700 grammi

B. Da 0 a 100 grammi

C. Da 1 a 2 kilogrammi

Quante ciglia ci sono nella palpebra inferiore?

A. Da 114 a 160

B. Da 0 a 6

C. Da 42 a 78

Dopo che l'acqua bolle qual è il tempo necessario per cucinare un uovo sodo?

A. Da 1 a 15 secondi

B. Da 5 a 10 minuti

C. Da 15 a 20 minuti

Qual è il peso di un coniglio (domestico)?

A. Da 3 a 8 kilogrammi
B. Da 101 grammi a 2 kilogrammi
C. Da 15 a 20 kilogrammi

Quanto è alto un semaforo (pedonale)?

A. Da 5 a 8 metri
B. Da 0 a 1 metri
C. Da 2 a 4 metri

Quanto tempo ci metti ad essere servito al banco del salumiere quando ci sono due persone prima di te?

A. Da 20 minuti a 3 ore
B. Da 10 a 15 minuti
C. Da 13 secondi a 5 minuti

Quanto è veloce una rondine in kilometri/orari?

A. Da 56 a 104 kilometri/orari

B. Da 1 a 8 kilometri/orari

C. Da 152 a 160 kilometri/orari

Quanto tempo ci metti ad allacciarti entrambe le scarpe?

A. Da 0 a 2 secondi

B. Da 18 secondi a 2 minuti

C. Da 4 a 10 minuti

Qual è il peso di un paio di scarpe eleganti?

A. Da 1 a 2 Kilogrammi

B. Da 101 a 200 grammi

C. Da 401 a 800 grammi
