# Supplementary material for: Proposal for a new tool assessing validity performance in forensic neuropsychological testing: the Test of Malingering in Abstraction Skills (TOMAS)
Source: Neurol Sci. 2025 Mar 3;46(6):2591–600. doi: 10.1007/s10072-025-08061-6 (PMC12084168; doi:10.1007/s10072-025-08061-6)
Supplement: Supplementary file 4 — Supplementary Material 4 [file 10072_2025_8061_MOESM4_ESM.pdf]

TOMAS Instructions

General presentation. This test evaluates your ability to make accurate estimates on some aspects of daily life. It consists of two parts (A and B) characterised by an increasing level of difficulty.

[the examiner presents the first part of the test by placing the administration material in front of the examinee; specific instructions for Part A are then provided. The examiner takes note of the examinee's answers using the notation sheet]

Part A. Below are some questions that require you to make an accurate estimate. Please choose the correct answer between the two alternatives, of which only one is correct, trying to be accurate in your estimate.

[at the end of the first part of the test, avoiding any feedback on performance, the examiner presents specific instructions for Part B]

Part B. Again, in this part of the test you will be presented with some questions on daily activities. However, here the level of difficulty will be greater as you are asked to identify the right answer among three alternatives, of which only one is correct, making an estimate as accurate as possible.

[the examiner presents the remaining questions of Part B one by one until the test is concluded; no help or clarifications are provided. The examiner takes note of the examinee's answers using the notation sheet]

Part A

Item	Alternatives	Correct answer	Provided answer	Score 0\1
1. What is the weight of a bar of soap?	A. 46 to 65 grams<br>B. 66 to 150 grams	B		
2. How long does it take to staple 10 copies of 3 pages of a newspaper?	A. 1 to 4 minutes<br>B. 5 a 6 minutes	A		
3. How long does it take for a washing machine to complete the wash program for bed sheets?	A. 41 minutes to 2 hours<br>B. 30 to 40 minutes	A		
4. How long does it take to close, write the address, and put the stamp on 5 letters?	A. 2 to 4 minutes<br>B. 5 to 10 minutes	B		
5. How many camels are there in Italy?	A. 4 to 27<br>B. 28 to 52	B		
6. What is the weight of a 90 by 45 by 60 cm aquarium (without water)?	A. 3 to 9 kilograms<br>B. 1 to 2 kilograms	A		
7. How long does it take to sew a button on a shirt?	A. 1 to 2 minutes<br>B. 3 to 5 minutes	B		
8 How much do heeled shoes weigh?	A. 280 to 520 grams<br>B. 112 to 279 grams	A		
9. How long is on average the spine of a man?	A. 49 to 91 centimeters<br>B. 92 to 133 centimeters	A		
10. What is the weight of a car's inner rearview mirror?	A. 401 to 650 grams<br>B. 151 to 400 grams	B		
11. What is the weight of a dining table chair?	A. 6 to 8 kilograms<br>B. 2 to 5 kilograms	B		
12. How long does it take a man to get a shampoo and a full haircut?	A. 31 to 55 minutes<br>B. 20 to 30 minutes	B		
13. How much water does it take to fill a bath?	A. 192 to 356 liters<br>B. 357 to 523 liters	A		
14. What is the maximum length of Sicily in kilometers?	A. 210 to 390 kilometers<br>B. 30 to 209 kilometers	A		
15. How much does a horse weigh?	A. 280 to 520 kilograms<br>B. 521 to 760 kilograms	A		
16. What is the weight of the rear bumper of a city car?	A. 1 to 2 kilograms<br>B. 3 to 9 kilograms	B		
17. What is the seating capacity on a 10-carriage train?	A. 504 to 936<br>B. 103 to 503	A		
18. Roughly how many coffees does the barman make in one hour during the rush hour at a motorway/highway service station ?	A. 105 to 195<br>B. 47 to 104	A		

Part B

Item	Alternatives	Correct answer	Provided answer	Schours 0\1
1. What is the weight of a common hair dryer?	A. 101 to 200 grams<br>B. 301 to 550 grams<br>C. 1 to 2 kilograms	B		
2. How many grams of pasta do you need to cook for 4 people?	A. 760 to 800 grams<br>B. 1 to 40 grams<br>C. 280 to 520 grams	C		
3. How many people are on a bus during the rush hour?	A. 171 to 200<br>B. 63 to 117<br>C. 1 to 9	B		
4. How long does it take a young man to walk one kilometer?	A. 7 to 13 minutes<br>B. 1 to 60 seconds<br>C. 19 to 20 minutes	A		
5. How long is a passenger carriage on a train?	A. 17 to 33 meters<br>B. 1 to 2 meters<br>C. 48 to 50 meters	A		
6. How long does it take to take a shower?	A. 2 to 5 minutes<br>B. 9 to 15 minutes<br>C. 30 to 31 minutes	B		
7. How much does a men's cotton t-shirt weigh?	A. 154 to 286 grams<br>B. 1 to 22 grams<br>C. 418 to 500 grams	A		
8. How long does it take for coffee to come out of a two-cup coffee maker?	A. 8 minutes to 3 hours<br>B. 7 seconds to 1 minute<br>C. 2 to 5 minutes	C		
9. How many matches are in a box?	A. 190 to 200<br>B. 1 to 10<br>C. 70 to 130	C		
10. How long does it take to wait for the traffic light to turn green?	A. 19 to 30 seconds<br>B. 1 to 3 minutes<br>C. 5 to 15 minutes	B		
11. How much is the surface area of a double bed sheet?	A. 2 to 5 meters^2<br>B. 0 to 1 meters^2<br>C. 6 to 7 meters^2	A		
12. How many weeks are there in one year?	A. 99 to 120<br>B. 1 to 5<br>C. 36 to 68	C		
13. What is the weight of a pair of medium-sized trousers (such as blue jeans)?	A. 301 to 700 grams<br>B. 0 to 100 grams<br>C. 1 to 2 kilograms	A		
14. How many eyelashes are there in the lower eyelid?	A. 114 to 160<br>B. 0 to 6<br>C. 42 to 78	C		

15. After the water boils how long does it take to cook a hard-boiled egg?	A. 1 to 15 seconds<br>B. 5 to 10 minutes<br>C. 15 to 20 minutes	B		
16. What is the weight of a rabbit?	A. 3 to 8 kilograms<br>B. 101 grams to 2 kilograms<br>C. 15 to 20 kilograms	A		
17. How high is a (pedestrian) traffic light?	A. 5 to 8 meters<br>B. 0 to 1 meters<br>C. 2 to 4 meters	C		
18. How long does it take to be served at the deli counter when there are two people before you?	A. 20 minutes to 3 hours<br>B. 10 to 15 minutes<br>C. 13 seconds to 5 minutes	B		
19. How fast is a swallow in kilometers/hour?	A. 56 to 104 kilometers/h<br>B. 1 to 8 kilometers/h<br>C. 152 to 160 kilometers/h	A		
20. How long does it take to tie both shoes?	A. 0 to 2 seconds<br>B. 18 seconds to 2 minutes<br>C. 4 a 10 minutes	B		
21. What is the weight of a pair of dress shoes?	A. 1 to 2 kilograms<br>B. 101 to 200 grams<br>C. 401 to 800 grams	C		
