# Supplementary material for: Proposal for a new tool assessing validity performance in forensic neuropsychological testing: the Test of Malingering in Abstraction Skills (TOMAS)
Source: Neurol Sci. 2025 Mar 3;46(6):2591–600. doi: 10.1007/s10072-025-08061-6 (PMC12084168; doi:10.1007/s10072-025-08061-6)
Supplement: Supplementary file 5 — Supplementary Material 5 [file 10072_2025_8061_MOESM5_ESM.pdf]

Test Of Malingering in Abstraction Skills

Instructions for print: print and staple the questions contained in the administration material on A4 sheets using horizontal layout.

TOMAS

Part A

What is the weight of a bar of soap?

A. 46 to 65 grams

B. 66 to 150 grams

How long does it take to staple 10 copies of 3 pages of a newspaper?

A. 1 to 4 minutes

B. 5 to 6 minutes

How long does it take for a washing machine to complete the wash program for bed sheets?

A. 41 minutes to 2 hours

B. 30 to 40 minutes

How long does it take to close, write the address, and put the stamp on 5 letters?

A. 2 to 4 minutes

B. 5 to 10 minutes

How many camels are there in Italy?

A. 4 to 27

B. 28 to 52

What is the weight of a 90 by 45 by 60 cm aquarium (without water)?

A. 3 to 9 kilograms
B. 1 to 2 kilograms

How long does it take to sew a button on a shirt?

A. 1 to 2 minutes

B. 3 to 5 minutes

How much do heeled shoes weigh?

A. 280 to 520 grams

B. 112 to 279 grams

How long is on average the spine of a man?

A. 49 to 91 centimeters

B. 92 to 133 centimeters

What is the weight of a car’s inner rearview mirror?

A. 401 to 650 grams

B. 151 to 400 grams

What is the weight of a dining table chair?

A. 6 to 8 kilograms

B. 2 to 5 kilograms

How long does it take a man to get a shampoo and a full haircut?

A. 31 to 55 minutes

B. 20 to 30 minutes

How much water does it take to fill a bath?

A. 192 to 356 liters

B. 357 to 523 liters

What is the maximum length of Sicily in kilometers?

A. 210 to 390 kilometers

B. 30 to 209 kilometers

How much does a horse weigh?

A. 280 to 520 kilograms

B. 521 to 760 kilograms

What is the weight of the rear bumper of a city car?

A. 1 to 2 kilograms

B. 3 to 9 kilograms

What is the seating capacity on a 10-carriage train?

A. 504 to 936

B. 103 to 503

Roughly how many coffees does the barman make in one hour during the rush hour at a motorway/highway service station?

A. 105 to 195

B. 47 to 104

TOMAS

Part B

What is the weight of a common hair dryer?

A. 101 to 200 grams

B. 301 to 550 grams

C. 1 to 2 kilograms

How many grams of pasta do you need to cook for 4 people?

A. 760 to 800 grams

B. 1 to 40 grams

C. 280 to 520 grams

How many people are on a bus during the rush hour?

A. 171 to 200

B. 63 to 117

C. 1 to 9

How long does it take a young man to walk one kilometer?

A. 7 to 13 minutes
B. 1 to 60 seconds
C. 19 to 20 minutes

How long is a passenger carriage on a train?

A. 17 to 33 meters

B. 1 to 2 meters

C. 48 to 50 meters

How long does it take to take a shower?

A. 2 to 5 minutes
B. 9 to 15 minutes
C. 30 to 31 minutes

How much does a men's cotton t-shirt weigh?

A. 154 to 286 grams

B. 1 to 22 grams

C. 418 to 500 grams

How long does it take for coffee to come out of a two-cup coffee maker?

A. 8 minutes to 3 hours

B. 7 seconds to 1 minute

C. 2 to 5 minutes

How many matches are in a box?

A. 190 to 200

B. 1 to 10

C. 70 to 130

How long does it take to wait for the traffic light to turn green?

A. 19 to 30 seconds

B. 1 to 3 minutes

C. 5 to 15 minutes

How much is the surface area of a double bed sheet?

A. 2 to 5 $meters^2$

B. 0 to 1 $meters^2$

C. 6 to 7 $meters^2$

How many weeks are there in one year?

A. 99 to 120

B. 1 to 5

C. 36 to 68

What is the weight of a pair of medium-sized trousers (such as blue jeans)?

A. 301 to 700 grams

B. 0 to 100 grams

C. 1 to 2 kilograms

How many eyelashes are there in the lower eyelid?

A. 114 to 160

B. 0 to 6

C. 42 to 78

After the water boils how long does it take to cook a hard-boiled egg?

A. 1 to 15 seconds

B. 5 to 10 minutes

C. 15 to 20 minutes

What is the weight of a rabbit?

A. 3 to 8 kilograms

B. 101 grams to 2 kilograms

C. 15 to 20 kilograms

How high is a (pedestrian) traffic light?

A. 5 to 8 meters

B. 0 to 1 meters

C. 2 to 4 meters

How long does it take to be served at the deli counter when there are two people before you?

A. 20 minutes to 3 hours

B. 10 to 15 minutes

C. 13 seconds to 5 minutes

How fast is a swallow in kilometers/hour?

A. 56 to 104 kilometers/hour

B. 1 to 8 kilometers/hour

C. 152 to 160 kilometers/hour

How long does it take to tie both shoes?

A. 0 to 2 seconds

B. 18 seconds to 2 minutes

C. 4 to 10 minutes

What is the weight of a pair of dress shoes?

A. 1 to 2 kilograms

B. 101 to 200 grams

C. 401 to 800 grams
